# Scanning European Needs and Expectations Related to Livestock Biosecurity Training by Using the World Café Method

**DOI:** 10.1155/2024/6743691

**Published:** 2024-03-30

**Authors:** Claude Saegerman, Jarkko K. Niemi, Nancy De Briyne, Wiebke Jansen, Alain Cantaloube, Marcel Heylen, Tarmo Niine, Julia Gabrielle Jerab, Alberto Allepuz, Ilias Chantziaras, Maria Rodrigues da Costa, Marie-France Humblet, Maria Eleni Filippitzi

**Affiliations:** ^1^Research Unit of Epidemiology and Risk Analysis Applied to Veterinary Sciences (UREAR-Uliège), Fundamental and Applied Research for Animal and Health (FARAH) Center, Department of Infections and Parasitic Diseases, Faculty of Veterinary Medicine, University of Liège, Liège 4000, Belgium; ^2^Bioeconomy and Environment Unit, Natural Resources Institute Finland (Luke), Seinäjoki 60320, Finland; ^3^Federation of Veterinarians of Europe (FVE), Brussels 1030, Belgium; ^4^Fédération Européenne pour la Santé Animale et la Sécurité Sanitaire (FESASS), Bruxelles 1000, Belgium; ^5^Dierengezondheidszorg Vlaanderen, Torhout 8820, Belgium; ^6^Institute of Veterinary Medicine and Animal Sciences, Estonian University of Life Sciences, Tartu 51006, Estonia; ^7^Veterinary Epidemiology Unit, Faculty of Veterinary Medicine, Ghent University, Merelbeke 9820, Belgium; ^8^Department of Animal Health and Anatomy, Universitat Autònoma de Barcelona, 08193, Cerdanyola del Vallès, Spain; ^9^Centre for Epidemiology and Planetary Health (CEPH), Scotland's Rural College (SRUC), Inverness Campus, Inverness, IV2 5NA, UK; ^10^Department of Occupational Protection and Hygiene, Unit Biosafety, Biosecurity and Environmental Licences, Liège University, Liège 4000, Belgium; ^11^Laboratory of Animal Health Economics, Faculty of Veterinary Medicine, Aristotle University of Thessaloniki, Thessaloniki 54124, Greece

## Abstract

The European Union Animal Health Law (2016/429) emphasizes disease prevention, underpinned by livestock biosecurity, surveillance, and traceability, as key aspects to minimize the risk of animal diseases. An important element of biosecurity is the training of key actors involved in implementing it. However, their needs and expectations regarding this training are poorly known. Under the COST action BETTER (CA20103), a World Café was organized to identify the needs and expectations of biosecurity training for farmers, veterinary practitioners, veterinary students, and other actors. A total of 78 participants distributed in four groups participated in the World Café. Needs and expectations were identified and ranked in decreasing order of importance. For farmers, the most important aspects were training focusing on practical aspects, the planning of training sessions in the day to accommodate workload, the need to prepare multiple reminders of upcoming training, and the short duration of events. For veterinary practitioners, it was considered that a mixed approach, including a theoretical and a practical part where people are invited to create a biosecurity plan and a follow-up report, were the most important features of training. For veterinary students, creating a good knowledge of the main principles of biosecurity was found as an essential element of training. Regarding other actors, gaining an understanding in the spread of pathogens and the repercussions on the cost of animal products that diseases might have (consumers), training on good/best practices of cleaning and disinfection and the development of clear protocols (transporters), and a mixture of formal and informal training and training on communication skills (other actors) were considered important. The World Café was a useful method to have a first identification, discussion, and differentiation on livestock biosecurity training needs and expectations of the key actors, although additional follow-up research involving more participants from more diverse countries with different coverage of cultures and education would be beneficial. These needs and expectations are relevant and should be considered when designing new training courses.

## 1. Introduction

The Animal Health Law (AHL) (Regulation (European Union (EU)) 2016/429) emphasizes disease prevention, underpinned by biosecurity, surveillance, and traceability, as key aspects to minimize the risk of animal diseases causing harm to society [1]. In the AHL, biosecurity is defined as “the sum of management and physical measures designed to reduce the risk of the introduction, development, and spread of diseases to, from, and within an animal population, or an establishment, zone, compartment, means of transport or any other facilities, premises, or location.” In a recent expert survey covering eight existing definitions of biosecurity, this definition was among the best-ranked definitions of biosecurity [2]. Only the conceptualization of the rule of five Bs (bio-exclusion, bio-containment, bio-compartmentation, bio-prevention, and bio-preservation) ranked higher in terms of expert elicitation (for more details, see [2, 3]).

**Figure 1:**
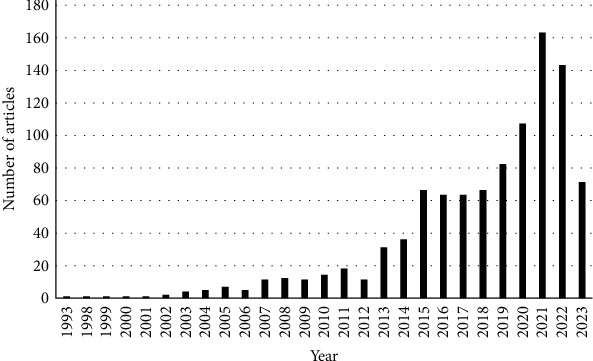
Number of publications (vertical axis) present in PubMed (US National Library of Medicine, National Institutes of Health) mentioning “livestock biosecurity,” by the year of publication (horizontal axis), 1998–2023 (*N* = 879). Data extracted on 23 July 2023.

Biosecurity is essential to prevent the introduction of pathogens into a farm and their spread inside and outside the farm [4]. Livestock biosecurity gained importance in the last decades (Figure 1). An important element of livestock biosecurity is the level of skills and the training of key actors involved in its implementation (e.g., [5, 6]). Training can be defined as “a planned learning experience designed to bring about permanent change in an individual's knowledge, attitudes, or skills” [7]. However, the needs and expectations of the key actors regarding livestock biosecurity training are not well known yet.

The BETTER COST action CA20103 (https://better-biosecurity.eu/) is dedicated to enhance biosecurity through training evaluation and raising awareness. For this purpose, it is essential to use participative approaches to better understand the factors affecting motivation and barriers for biosecurity implementation so that evidence-based effective communication strategies can be developed for different groups of actors.

This study addresses the knowledge gap regarding the training needs of different stakeholders on livestock biosecurity. The aim of this study was to identify and rank, in a decreasing order of importance, the needs and expectations of four groups of key actors concerning livestock biosecurity training. The World Café, an innovative method to facilitate reflections of people [8], was used to capture needs and expectations related to livestock biosecurity training. The groups of interest were (i) veterinary practitioners, (ii) farmers, (iii) veterinary students (academic curricula, specialization), and (iv) other actors (e.g., traders, private companies, general population, and international bodies, etc.).

## 2. Materials and Methods

### 2.1. The World Café Method

The World Café is an inspiring and flexible participatory process to explore emerging themes or topics, to collect best practices or suggestions, to generate improvements or recommendations, and to define priorities for the implementation of projects or research agendas [9]. The method is used in various settings such as community health service [10], consumer service [11], manufacturing [12], research prioritization [13], activity planning, and elicitation of community group perspectives [14], plant and public health program evaluation and planning [13, 15, 16], and animal health [17]. Briefly, it is a flexible approach to facilitate group discussions that can be used to engage stakeholders and encourage participation and constructive dialogs while discussing a specific topic [14, 18]. Many operational resources are freely available on how to set up this method [19].

**Figure 2:**
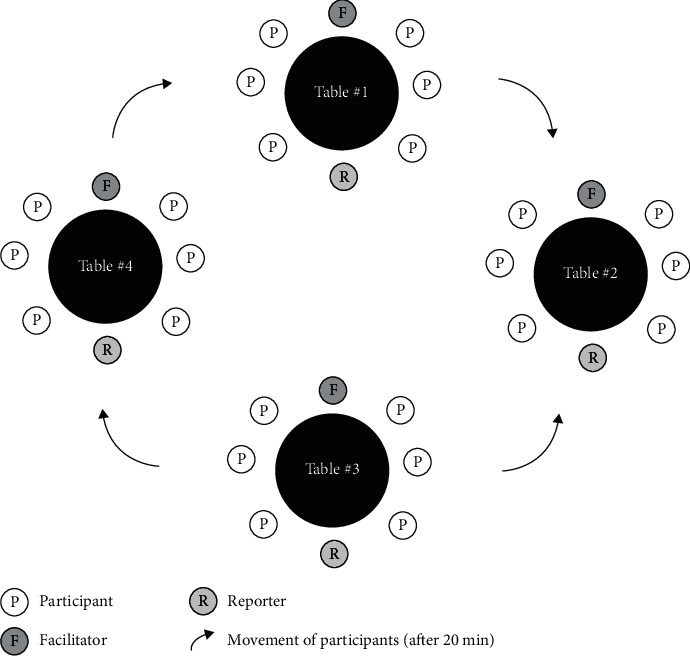
The World Café setup.

A World Café discussion covers several topics (each with an assigned facilitator and a reporter) that are discussed by small groups of participants rotating between the topics (Box 1 and Box 2). Efforts toward progress are pursued during each rotation, aided by the facilitator, who provides a brief introduction at the beginning of each round to update the new group about the contributions made by the preceding groups [20] (Figure 2). In the original World Café format, special effort is made to encourage informal discussions, making participants feel at ease and creating a relaxed discussion environment resembling a Café [17, 20].

### 2.2. Study Design and Participant Selection

The World Café was conducted on 7 February 2023 in the Ghent University Museum (Belgium) during a 1-day workshop on the BETTER COST action. Before the World Café, all the participants followed a plenary session that focused on challenges on implementation of biosecurity and training needs, with views presented by experts from the European Commission (Jean-Charles Cavitte, European Commission), the field veterinarians (Nancy De Briyne, Federation of Veterinarians of Europe), the farmers (Alain Cantaloube, Fédération Européenne pour la Santé Animale et la Sécurité Sanitaire (FESASS)) and academia (Jeroen Dewulf, Ghent University).

**Figure 3:**
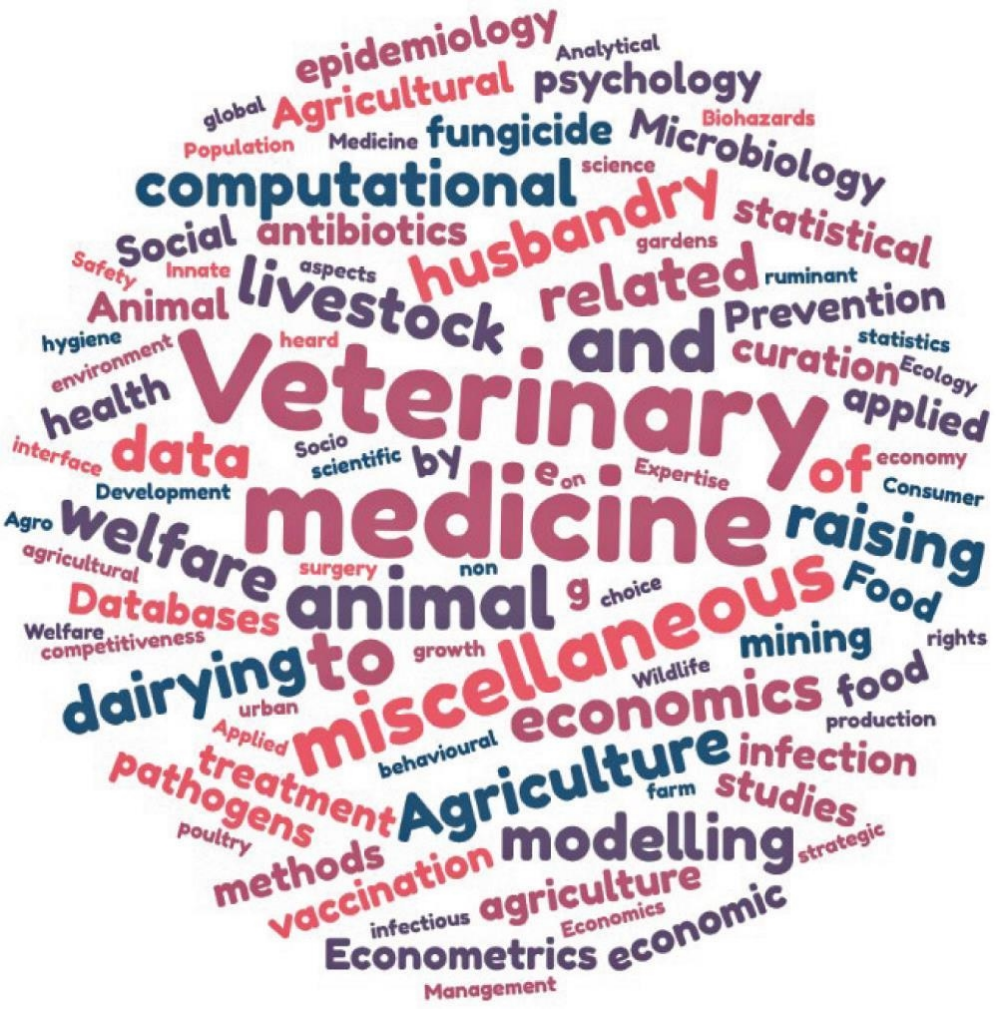
Expertise of participants from the BETTER COST action. Respectively 39, 11, 9, 4, 7, 5, 3 participants coming from universities, were students, coming from institution, were veterinary practitioners or representatives, were farmers or representatives, coming from private companies, or coming from international bodies.

**Figure 4:**
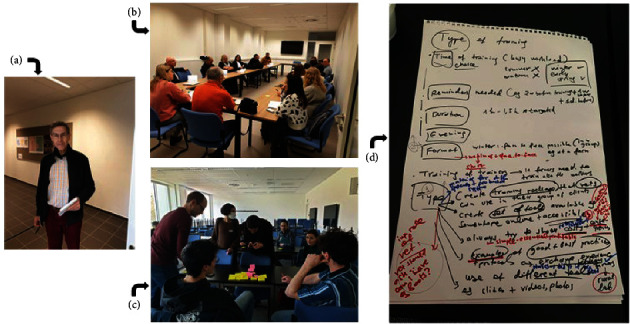
The coordinator of the World Café served as timekeeper (a) and did not intervene in the discussion tables for which a facilitator and a reporter were assigned (b and c) so that novel ideas (d) were generated and documented in a structured manner.

A total of 78 participants to the COST BETTER (veterinarians with different expertise, farmers and their representatives, students, and other stakeholders) (Figure 3) were involved in the World Café with four tables of discussion in order to bring about their needs and expectations related to livestock biosecurity training (Figure 4). Each table focused on one type of actors (i.e., veterinary practitioners and their representatives, farmers and their representatives, veterinary students, and other actors). At the end of each discussion, the participants pointed out the most important needs and expectations by the targeted actors in their table and ranked them. In each table, the discussion was supported by a facilitator who gave a short introduction at the start of each rotation to inform the incoming group about the input of previous groups. In addition, a reporter was assigned to each table to share information about the discussions with all participants at the end of the World Café. A strength of the World Café was the participation of the relevant stakeholders in the respective groups (so not only scientists but also, for example, students in the students' group and farmers in the farmers' group).

### 2.3. Practical Recommendations

General recommendations, timing (Box 1), and specific recommendations for facilitators (Box 2) were presented to all participants in a plenary session.

## 3. Results

The main needs and expectations are summarized and depicted per group of actors involved in livestock biosecurity training: private veterinarians (Table 1), farmers (Table 2), veterinary students (Table 3), and other actors (Table 4).

## 4. Discussion

The main finding of the study is that biosecurity training needs differ from one actor to another because they have different levels of scientific/practical knowledge, different workloads, and different needs. The information collected is relevant whenever designing training and education programs in livestock biosecurity.

Both individual approaches, such as the interview of an expert, or approaches involving the interaction between the study participants, such as Delphi, benchmarking, focus group discussion, and the World Café methods, exist to collect data or ideas from people or a group of people [21]. In this study, the World Café method was applied because it is an appreciated and inspiring method that allows collaborative conversations, hence stimulating creativity and collective wisdom and sharing practical knowledge [9, 21]. While in some World Café studies, the consensus and dissensus are assessed (e.g., [17]), this assessment was not performed in this study because the objective of the workshop was to achieve a collective listing and ranking of needs and expectations related to livestock biosecurity training.

Another parameter sometimes followed in a World Café is the level of saturation of ideas [22]. According to each group facilitator and reporter of this World Café, a certain degree of saturation [23] was achieved after the passage of four groups of participants at each table (topic). This may be related to the number of participants or groups of participants involved in the World Café, the good mixing of participants per group, the countries of origin of participants, and/or a good animation permitting the active and balanced participation of each participant.

The following elements describe the most important needs and expectations for biosecurity training as resulting from the World Café. Whatever the group of actors, the main and commonly believed need was related to promoting a mixed approach that includes both theoretical and practical training on livestock biosecurity. Education and training programs designed with more interactive (ideally face-to-face), communicative, and participatory approaches had a positive impact on the assimilation of information and the effective implementation of control strategies [24]. For farmers, for example, resolving problems, a proper selection of methods and a good balance between practical (70%) and theory (30%) were recommended [25].

In a WOAH survey, most of the country participants requested farm biosecurity support training [26]. For farmers, according to the World Café, the strategical calendarization of training and its short duration were believed to be important due to the heavy workload. Several reminders of key aspects of biosecurity were also thought to be useful. These findings agree with the agricultural trainings' guidelines, which recommend to choose the right time and the best length for trainings, depending on the needs and availability of farmers, which naturally depend on their workload and duties but also on their household responsibilities. For instance, individuals may have varying availability based on their daily responsibilities, with some potentially having heavier involvement in household duties like childcare or other activities. This can be influenced by individual circumstances rather than gender assumptions [27]. Furthermore, the practice of repeating training was previously acknowledged as a strategy to enhance the implementation of livestock biosecurity measures [28]. Practicing a task repeatedly is useful to learn a new skill and to increase its performance [29]. In addition, an experience-based (rather than exposure-based) training protocol may allow a better transference of skills to related tasks [30].

According to the World Café, creating a good knowledge of the main principles of livestock biosecurity was a basic-essential requirement for veterinary students. This need was previously listed in the WOAH (OIE) Global Conference on Evolving Veterinary Education for a Safer World [31] and included in the Veterinary Education Core Curriculum WOAH Guidelines [32]. The main principles of livestock biosecurity are included in different book chapters (e.g., [33, 34]) as well as biosecurity standard operating procedures (e.g., [35]).

For transporters, training on best practices of cleaning and disinfection of vehicles and the development of clear protocols were believed to be the most important needs. Standard operating procedures for transport biosecurity include cleaning and disinfecting the vehicle that has transported the live animals and preventing the transporter from entering barns to avoid contact with animals on the farm [36]. As an example, the process of cleaning and disinfection of animal transport vehicles after unloading animals at the abattoir is an important step related to biosecurity and a critical control point regarding proper hygiene [37]. A recent German survey in five abattoirs (750 vehicles included) indicated an important margin of improvement as, depending on the abattoir, 31%–97% of all vehicles were only cleaned and as little as 3% up to a max of 59% were both cleaned and disinfected [37]. In order to engage stakeholders in livestock biosecurity, their integration is needed in the assessment, management, and communication of risks concerning animal health and biosecurity [38].

Consumers as stakeholders need to understand the role that livestock biosecurity can play in the prevention of the spread of pathogens and its repercussion on the cost of animal products that diseases might have. Indeed, the decision to implement biosecurity measures is partially related to the risk of the disease faced by farms [39, 40]. The two components of risk to be considered include the likelihood of disease occurrence and the severity of disease consequences [41]. According to the WOAH, 60% of pathogens that cause human diseases originate from domestic animals or wildlife, 75% of emerging infectious human diseases have an animal origin, and 80% of pathogens that are of bioterrorism concern originate from animals [32]. Costs of diseases are a high concern, and net financial costs associated with an epidemic of both zoonotic and non-zoonotic animal diseases and vector-borne diseases may be substantial. To illustrate the costs of diseases, some examples of various species are proposed hereafter. Indeed, the 2001 foot-and-mouth epidemic in the United Kingdom generated losses of €193 million for sheep farmers [42]. In Niger, at the cattle herd level, the economic impact of foot-and-mouth disease was estimated at €499 [43]. In the Netherlands, losses reached €24.75 million and €1 million for the ovine and the caprine sectors, respectively, during the 2007 bluetongue epidemic [44]. Indeed, sheep can develop severe clinical signs and die from the disease [45]. For the Walloon Region (southern part of Belgium) and for the period 2006–2007, the average technical–economic losses due to the bluetongue (serotype 8) in cattle and small ruminants were estimated at €93 million [46]. The cost for the US industry due to the porcine epidemic diarrhea virus was estimated between $900 million and $1.8 billion from 2013 to 2014 [47, 48]. A recent evaluation of the direct cost of African swine fever outbreaks showed a wide variation between countries: US$ 826,911 in Vietnam, US$ 6,196,760 in North Macedonia, and over US$ 58 million in the Philippines [49]. Total losses associated with the 2007–2011 Q fever outbreak that occurred in the Netherlands were estimated at €307 million, along with a human burden of 2,462 disability-adjusted life years [50].

When considering the delivery of training, a mixture of formal and informal training was proposed by the participants of the World Café, and some of them mentioned the following tools: “on demand” video capsules, leaflets, infographics, radio podcasts, and TV-program learning. According to Noe [51], training effectiveness is determined by four levels of training outcomes: (i) trainees' reactions to the program content and training process (reaction); (ii) knowledge or skill acquisition (learning); (iii) behavior change (behavior); and (iv) improvements in tangible individual or organizational outcomes such as turnover, accidents, or productivity (results). Moreover, motivation and environmental conditions influence the training effectiveness [51]. An effective training is possible when the competency, practice-oriented approach, and active and interactive teaching methods are used [24, 52]. Although formal learning is the main route to recognized training qualifications required to enter certain jobs, informal learning acquired through experience in work and life is the most frequently used form of learning [52]. Combining formal, nonformal, and informal learning was recommended to develop workforce skills [52]. Several innovative teaching methods have recently been developed, and some are cited in the following sentences as examples. According to the literature, game elements (gamification) might increase participants' cognitive engagement and change of their priorities or strategies during learning. Game elements induce better training performance but comparable learning gains as nongame-based training group [53]. For livestock biosecurity, recent game elements [54] and digital extension interactive voice response [55] were proposed in order to assess strategic, tactical, and operational decision-making and risk in a livestock production chain [56]. Despite some evidence suggesting that innovative training programs are effective in improving the performance of health workers [57], more studies to assess properly the impacts of training methods on the biosecurity skills, knowledge, behavior, and attitude change of trainees are needed.

Regarding other actors (producers, advisors, managers, policymakers, veterinary statutory bodies, wildlife managers, gamekeepers, and hunters), training to develop communication skills was emphasized. Despite the increasing emphasis on the teaching and assessment of communication skills [58] and a clear demonstration of the advantage of interactions between these actors (farmers and private veterinarians regarding livestock biosecurity), there is also a need to evaluate the existing communication-specific training received by these persons and hence to explore to what extent suboptimal communication skills negatively impact the uptake of biosecurity practices [59].

The results of this study give valuable first information on needs and expectations related to livestock biosecurity training in the EU. However, more extended research involving more participants from the different actors/countries inside and outside the EU, with different coverage of cultures and education, is recommended. To scale the study up to other continents, more participants originating from outside Europe should be engaged. To obtain relevant additional information on training needs, a survey on existing trainings per group of actors (farmers, veterinarians, veterinary students, and other actors) is recommended. We can also use the findings of this study to redesign biosecurity training programs in a proper way.

This study also highlighted the balanced request of both principles and practical aspects of livestock biosecurity, including the assessment of biosecurity compliance and the improvement/implementation of biosecurity action plans [56, 60]. This is in line with the FAO effort on the progressive management pathway for animal biosecurity [61].

## 5. Conclusions

Livestock biosecurity has been deemed pivotal by the EU AHL [62] and by the FAO by the Progressive Management Pathway for Terrestrial Animal Biosecurity (FAO-PMP-TAB) [61]. In this study, the World Café was a useful method to discuss, identify, and rank the needs and expectations of different categories of actors related to livestock biosecurity training. Different needs and expectations were highlighted for each actor, revealing a multifaceted challenge for the aspiring biosecurity trainers. Indeed, a mixture of motivating, practical, and theoretical training is needed to ensure that the biosecurity skills of trainees are enhanced effectively.

## Figures and Tables

**Box 1:**
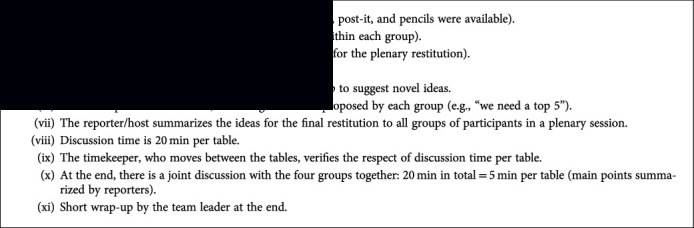
General recommendations and timing of World Café.

**Box 2:**
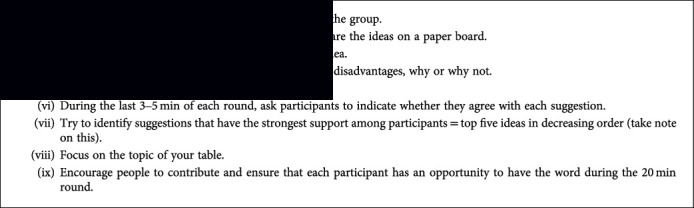
Specific recommendations for facilitators of the COST BETTER World Café.

**Table 1:** Main needs and expectations of veterinary practitioners related to livestock biosecurity training.

Facilitator: Nancy De Briyne (FVE)

Reporter: Wiebke Jansen (FVE)

Main needs and expectations in decreasing order of importance based on times mentioned by the group:
(i) A mixed approach: training course contents with theory and principles, followed by a practical part where people are invited to make biosecurity plans and report afterward. (ii) Veterinarians need to evaluate the costs and benefits of specific biosecurity training before undertaking it. (iii) Soft skills in communication and behavioral change are needed to promote the veterinarian-farmer dialog. (iv) Practices/veterinarians that are trained in biosecurity need to be recognizable (label or certificate) and/or to be counted as continuing professional development. (v) Competent authorities and public veterinarians should also be involved in training. (vi) Staged training (some initial, some continuous training in a flexible way) is needed. (vii) Use “ambassadors network” so that people who have been trained on biosecurity pass on their knowledge to other stakeholders. (viii) Veterinarians should know that, according to the Animal Health Law, they need to give advice on biosecurity.

**Table 2:** Main needs and expectations of farmers related to livestock biosecurity training.

Facilitator: Alain Cantaloube (FESASS) and Marcel Heylen (DGZ)

Reporter: Maria-Eleni Filippitzi (BETTER COST action member)

Main needs and expectations in decreasing order of importance:
(i) Practical aspects: The calendarization of the training is important due to variations in the farmers' seasonal workload. It is essential to provide multiple reminders of upcoming training sessions, and the duration of each session should be brief, ideally not exceeding 1–1.5 hr. (ii) Format: Webinars (short and possibly hybrid) and face-to-face meetings (as long as the practical aspects above are met). Farm visits could be an option, but they include risks (e.g., biosecurity, possible pathogen transmission). The creation of a set of tools (e.g., presentations, videos, photos) that could be available online on a dedicated webpage and easily accessible has also been suggested. (iii) Content: Use examples of good and bad practices and protocols, show the costs of interventions, return on investment, and the importance of biosecurity in relation to its consequences (including the consequences of not implementing appropriate biosecurity), and keep the information simple and essential. (iv) The importance of veterinarians was highlighted. Therefore, the training of veterinarians on biosecurity is important too. The need to train vets (and farm workers) was highlighted. (v) How to approach and motivate farmers? Through (i) farm veterinarians, (ii) the support of farmers associations, (iii) a community of practices where the needs of each sector and type of farming are understood, and (iv) the exchange of experiences and success stories between farmers (“I did it and it worked”).

**Table 3:** Main needs and expectations of veterinary students related to livestock biosecurity training.

Facilitator: Tarmo Niine (CA20103 member)

Reporter: Julia Gabrielle Jerab (veterinary student and BETTER COST action member)

Main needs and expectations in decreasing order of importance:
(i) To create a good knowledge of the main principles of biosecurity. Although veterinary medicine is constantly changing, and there are many different livestock sectors, the main pillars of biosecurity remain the same. It is essential to make the students think in a bio-secure way. (ii) Hands-on application of biosecurity principles. Farm visits, role-playing in class, and solving cases that stimulate active thinking and application of biosecurity. (iii) To stimulate problem-solving and analytical skills. To promote creativity in solving biosecurity problems. (iv) To learn how to communicate with farmers based on their priorities, how to understand what farmers want and how to motivate them to implement biosecurity measures. (v) To integrate biosecurity training throughout the veterinary education curricula. Biosecurity is a piece of a larger puzzle. Students should understand the essential role it plays in all aspects of veterinary medicine.

**Table 4:** Main needs and expectations of other groups of actors related to livestock biosecurity training.

Facilitator: Jarkko Niemi (BETTER COST action member)

Reporter: Alberto Allepuz (BETTER COST action member)

Main needs and expectations in a decreasing order of importance:
Group 1—Consumers
(i) To understand the role they can play in the spread of pathogens and the repercussion disease can have on the cost of animal products that diseases might have.

Group 2—Producers, advisors, managers, policymakers, and veterinary statutory bodies
(i) To improve communication skills. (ii) Training on the economics so they can include the costs and benefits of biosecurity in their decision-making process. (iii) To understand the feasibility of biosecurity measures on different production systems. (iv) To understand the contributions of service providers (e.g., pest control, repairing service providers in farms). (v) To understand the principles of biosecurity and their role in the spread of pathogens.

Group 3—Transporters
(i) Training on best practices of cleaning and disinfection, the development of clear protocols for biosecurity. (ii) To understand the basics of biosecurity to make transporters comprehend their contribution to the spread of pathogens between farms.

Group 4—Wildlife managers, gamekeepers, and hunters
(i) Training on communication skills. (ii) Training on biosecurity measures that can prevent the transmission of pathogens between domestic animals and wildlife.

Group 5—All target groups: delivery of training
(i) The most preferred method of training will depend on the type of stakeholder. In general, a mixture of formal and informal training was recommended. (ii) Mentioned tools included, among other methods, short videos, leaflets, infographics, radio, TV-program, and E-learning. (iii) The importance of developing practical trainings to see how things work in a face-to-face format whenever it is possible was mentioned.

## Data Availability

The data that support the findings of this study are presented in this publication.

## Additional Points

*Limitation of the Study:* The representativity in epidemiology and the saturation of information in sociology are not in opposition. Both permit inference. Sample representativeness is the degree to which a sample “looks like” the true population on some set of characteristics. Data saturation is the degree to which adding respondents to the sample will not include additional novel information [63]. Data saturation is frequently used when a sampling frame is not available (the case of this exploratory study). While a first list of needs and expectations was obtained by this World Café, the scaling of the study to other continents was proposed to capture better the difference in education and culture. In addition, other possibilities of statistical analysis of the data exist (e.g.) and should be the inclusion of nonparametric statistical techniques like those used in expert elicitation (e.g., [64–66]) or to submit the list of needs and expectations captured during this World Café to representative stakeholders for voting the relative importance of the different needs and expectations (e.g., [2]).
